# A Mixed-Methods Evaluation of a Virtual Rehabilitation Program for Self-Management in Post-COVID-19 Syndrome (Long COVID)

**DOI:** 10.3390/ijerph191912680

**Published:** 2022-10-04

**Authors:** Thuvia Flannery, Hannah Brady-Sawant, Rachel Tarrant, Jennifer Davison, Jenna Shardha, Stephen Halpin, Manoj Sivan, Denise Ross

**Affiliations:** 1 Leeds Long COVID Rehabilitation Department, Leeds Community Healthcare Trust, Leeds LS12 5SG, UK; 2 Department of Rehabilitation, Leeds Teaching Hospitals NHS Trust, Leeds LS1 3EX, UK; 3 Academic Department of Rehabilitation Medicine, University of Leeds, Leeds LS1 3EX, UK

**Keywords:** virtual course, SARS-CoV-2 virus, post-acute sequalae of COVID-19, digital technology, Multi-Disciplinary Team, Template Analysis

## Abstract

Long COVID (LC) symptoms can be long standing, diverse and debilitating; comprehensive multidisciplinary rehabilitation programs are required to address this. A 10-week LC Virtual Rehabilitation Program (VRP) was developed to provide early education and self-management techniques to address the main symptoms of LC and was delivered to a group of persons with Long COVID (PwLC) online, facilitated by members of the multi-disciplinary rehabilitation team. This paper describes an evaluation of this VRP. Questionnaires completed by Healthcare Professionals (HCP) delivering the VRP were thematically analyzed to gain a priori themes and design semi-structured telephone interview questions for PwLC. Template analysis (TA) was used to analyze interview data. Routinely collected patient demographics and service data were also examined. Seventeen HCP survey responses were obtained and 38 PwLC telephone questionnaires were completed. The HCP interviews generated three a priori themes (1. Attendance and Availability, 2. Content, 3. Use of Digital Technology). TA was applied and three further themes emerged from the combined HCP and PwLC responses (4. Group Dynamics, 5. Individual Factors, 6. Internal Change). Key outcomes demonstrated that: the VRP was highly valued; digital delivery enabled self-management; barriers to attendance included work/life balance, use of technology, health inequalities; and LC was poorly understood by employers. Recommendations are provided for the design of VRPs for LC.

## 1. Introduction

Post-acute sequalae of SARS-CoV-2, post COVID-19 condition [1] and post-COVID-19 syndrome, commonly known as Long COVID [2] (LC) in the United Kingdom (UK), has placed significant additional demand on the UK National Health Service (NHS) [3] and on global healthcare systems. This is due in part to the complexity of the multi-system involvement and diverse range of debilitating symptoms, in addition to high prevalence, and lack of understanding of pathophysiology and definitive treatments of the condition. 

UK data to June 2022 estimated that 2 million people, approximately 3% of the UK population, were experiencing self-reported LC symptoms, that is, continuing symptoms for more than four weeks after first suspected COVID-19 infection [3]. This is independent of severity of infection or hospitalization [4]. Long COVID symptoms negatively affect the day-to-day activities of 1.4 million people (72% of people with LC) [4].

Commonly reported LC symptoms include fatigue, breathing issues (shortness of breath), ‘brain fog’ (cognitive impairment), depression and anxiety, palpitations, dizziness, insomnia, dietary issues, and joint pain [1,2,3,4]. In addition, many Persons with Long COVID (PwLC) may experience symptoms that vary in intensity, do not follow a linear path of recovery and can be fluctuant, with relapse triggered by activities involving physical or cognitive exertion [1,5]. 

Since March 2020, the UK NHS has been forced to accelerate the adoption of digital technology [6]. This was to limit face-to-face encounters and so the transmission of COVID-19, aiming to empower patients to self-manage their condition(s) to reduce demand on primary care and emergency services [6,7]. This change is supported by evidence from several rehabilitation studies, where traditional healthcare was optimized by e-health interventions to enhance safety, efficacy, and treatment adherence. These demonstrated comparable patient outcomes between face-to-face and virtual rehabilitation interventions [8,9,10,11,12,13,14,15]. The principles of virtual rehabilitation synthesised from several studies and systematic reviews in stroke, cardiac and pulmonary rehabilitation were applied to the development of the Leeds LC Rehabilitation Service during the acute phases of the pandemic [8,9,10,11,12,13,14,15]. During this timeframe the service has designed, implemented and evaluated their virtual interventions. 

In their review of the literature supporting the use of modern technologies in post-COVID-19 cardiopulmonary rehabilitation, Andrtoi et al. [16] present the use of previously established programmes such as cardiac rehabilitation; hybrid programmes such as part face to face and part virtual; use of digital video disc; home based and telephone consultations; E-learning programmes; interactive video consultations to monitor patients in real time. All this work is in support of rehabilitation for PwLC. Furthermore, two studies describe the feasibility of tele-rehabilitation programmes for PwLC [17,18]. The authors feel that the VRP as a contribution to the overall delivery of the LC rehabilitation service meets the requirements suggested by Pinto et al. [19].

Ongoing conversations between Healthcare Professionals (HCPs), PwLC, carers and researchers in addition to reviews of service delivery ensured that the needs of the LC population were recognised, supporting best available practice and the growing evidence base [20,21,22].

The Leeds LC Rehabilitation Service (Team) as of July 2022 has an active caseload of 1233 with a waiting list of 178 patients. The longest wait for a telephone triage appointment is nine weeks, with an additional wait of between 14 and 22 weeks for a face-to-face appointment, depending on the profession. The Team receives between 80 and 140 referrals per month and have been delivering a virtual rehabilitation programme (VRP), alongside face-to-face interventions if required since December 2020. The VRP format consists of weekly hour-long sessions, at a set time over 10-weeks on Microsoft Teams (MT). Each session has a pre-recorded video lasting approximately 20 min, with the remainder of session reserved for peer discussion. The peer discussion in sessions is facilitated by two members of the Multi-Disciplinary Team (MDT) and group size can vary from 20 to 40 PwLC. Each session focuses on one of the key symptoms of LC [23,24] (see Table 1) and the VRP aims to provide an interactive overview of these symptoms, enabling a self-management approach. These sessions were developed from the experience of the MDT of working with PwLC, and the symptoms they report. 

Most online courses are provided on a platform for individuals to work through at their own pace with separate access to health professionals and forums to provide peer support and opportunities for questions [8,12]. The VRP offers attendance and support within the same peer group with real time facilitated interaction. It is suggested that this approach of supported self-management, online education and peer support increases the chances of adoption of behaviours which positively contribute towards participant’s health [6,21,23,24]. 

The Leeds LC team have consistently evaluated their service and rehabilitation intervention for PwLC [20,21,22,23]. As little is known about how digital interventions may contribute to the health and wellbeing of PwLC, an evaluation of this VRP was considered important in terms of inclusivity, best evidence, and quality-driven healthcare delivery. 

The aims of this service evaluation were to gather feedback from HCPs and PwLC, to understand their needs and inform future VRP development. The applied principles of mixed methods design are supported by published evaluations of telemedicine in several long-term conditions and was considered an appropriate approach in healthcare topics where little evidence exists [25,26]. 

## 2. Materials and Methods

### 2.1. Design

A pragmatic, consecutive mixed methods design was used to evaluate the VRP. Two separate questionnaires, one for HCP and one for PwLC, were developed using both open (qualitative), and closed (quantitative) questions to obtain comprehensive feedback from those delivering the VRP (HCP) and those participating (PwLC). Attendance rates and the demographics of gender, age, ethnicity and duration of LC symptoms were also collected. 

### 2.2. Setting

A community healthcare NHS Trust within the North of England, UK.

### 2.3. Recruitment

People with LC, who had previously consented to participate in service review projects, were asked to provide feedback on their impressions of the VRP.

### 2.4. Data Collection

The first questionnaire was directed at HCP delivering the VRP (Supplementary Figure S1). This was informed from the VRP monthly clinical team meetings and then sent via email using Microsoft Forms with an anonymous reply. Qualitative and quantitative responses were recorded using Excel. The second questionnaire was directed at PwLC (Supplementary Figure S2). This was developed using the responses that emerged from the questionnaire given to the HCPs. The Clinical Research Fellows (CRFs) telephoned PwLC VRP participants to complete the questionnaire using a standardised script (further explanations and questions were allowed to enable PwLC to understand the questions and give full answers); qualitative responses were recorded using rich text, and quantitative data were recorded using Excel. Questionnaires were completed within two to three weeks of completion of the 10-week VRP to reduce recall bias. Telephone contact was used as opposed to email to include those who may experience difficulty with technology and thus maximise responses. 

### 2.5. Analysis

The qualitative data from both questionnaires were combined. Template Analysis was used to thematically analyse the data, using the a priori themes and creating new themes as they emerged (Supplementary Table S1) [27]. Using template analysis allowed a pragmatic and flexible means of developing and organising the themes emerging from several sources of data, i.e., from the evidence base [8,9,10,11,12,13,14,15], and the scripts recorded from the questionnaires completed by PwLC and the HCPs. The initial template was created using themes arising from the outcomes of the questionnaire sent to the HCPs. The Template coding process and emergent themes were reviewed and re-reviewed by the research team during discussion within regular research group meetings. This ensured on-going reflexivity and reduction in the risk of bias. Relevant demographics and aspects such as perceived attendance rates were examined using Microsoft Excel. 

### 2.6. Inclusion

All HCPs who were responsible for delivering the VRP were invited to participate. People with LC who had previously consented to participate in service evaluation projects and who also attended the VRP were invited to participate. 

### 2.7. Exclusion

People who were unable to read or understand spoken English were excluded from this service evaluation (n = 0). Those who had not consented to participate in service evaluation were also not included (n = 2). 

### 2.8. Ethics

Approval for this service evaluation was granted by The Leeds Community Hospitals NHS Trust R&I Department: LCH Ref: SE/0132. The decision tool provided by UK NHS Health Research Authority (HRA) website indicated that the processes used within this work were not considered to be research and so did not require separate HRA ethical approval. 

## 3. Results

Seventeen out of 20 HCPs delivering the VRP responded to the HCP questionnaire: including five Occupational Therapists, eight Physiotherapists, one Dietician and three professionals in the “other” category. Two cohorts of PwLC (n = 38) responded to the invite to participate with the PwLC questionnaire (Table 2). These people had previously consented to being contacted for service evaluation or research purposes, and had been assigned to the VRP during the evaluation timeframe between November 2021 and March 2022. 

### 3.1. Quantitative Data Analysis

Twenty-one PwLC had previously used Microsoft Teams (MT), 14 had not previously used and three did not answer. Sixteen reported issues with MT: six with the Link, six because they were new to technology and four related to Wi-fi issues. We found no correlation between duration of LC symptoms, severity of symptoms, age or gender.

Group size was not significant with only three PwLC stating that the group size was ’too big’ and no-one reporting ‘too small.’ Current times and days of week that the VRP sessions were available were acceptable to 24 people, although six would have preferred later in the morning, five afternoon sessions, two in the evening and one PwLC preferring a weekend delivery. 

An important aspect of the VRP evaluation were the views of PwLC on their ability to put the knowledge and skills from the VRP into practice, and the barriers they encountered. Thirty-six felt that they had been able to, one stated that they had not been able to achieve this, and one did not answer. Barriers to implementing these skills were work (n = 3) and home (n = 1).

**Figure 1:**
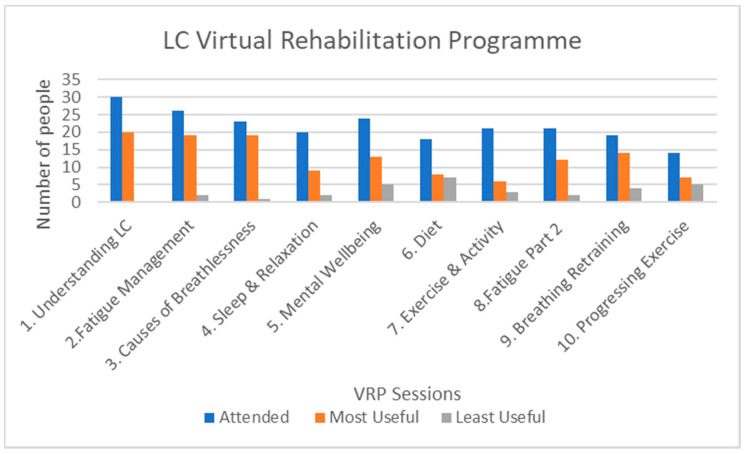
Sessions attended: Most and Least useful session(s).

VRP sessions had variable attendance some being better attended than others and this is reflected in ‘most useful’ and ‘least useful’ sessions (Figure 1). Ten PwLC attended less than 50% of sessions with the following reasons given: Work/Life (n = 5); Format/Content (n = 2); Technical issues/Link (n = 2) and no IT knowledge (n = 1).

### 3.2. Qualitative Data Analysis 

**Figure 2:**
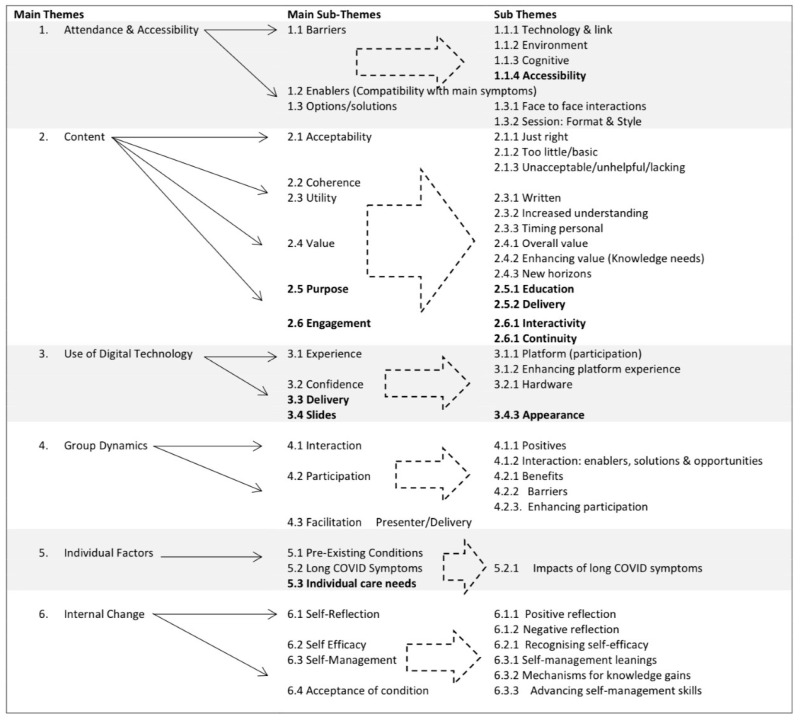
Template Analysis combines both HCP and PwLC data (abridged).

Figure 2 displays Main themes, Main Sub-Themes and Sub-Themes of the combined TA. Within the evaluation, the sub-themes were further broken down but are not discussed in this paper, themes indicated in bold were HCP only themes. All other themes emerged from both HCPs and PwLC.

#### 3.2.1. Main Theme 1: Attendance and Accessibility

This a priori theme included main sub-themes of **Barriers (1.1)** and **Enablers (1.2).** These focused on several sub-themes around technology and access to technology (sub-theme **Technology link 1.1.1**), associating the influence of environment when attending the VRP and cognitive issues related to LC (sub-theme **Cognitive 1.1.3**), “*Difficulty with link so couldn’t join*” (PwLC), “*had issues logging on initially when enrolled onto two courses, now enrolled onto a third course and IT issues have resolved*” (PwLC). Other comments included “*A chance for those who can afford the device, have the IT skills and are time rich to learn more to aid their rehabilitation*” (HCP). Reference was made to condition specific difficulties with an online delivery “*Links can be more cognitively challenging—need to have value indicated*” (PwLC). The barrier of **Environment (1.1.2)** emerged, for example “*Tricky as I don’t think I committed to it due to work*” (PwLC). Further identified barriers included “*Fitting into work time*” (PwLC) and “*Work/Life demands*” (PwLC) in addition to “*I was unable to attend any of the others due to home circumstances*” (PwLC). 

Main sub-themes **Enablers (1.2)** and **Options/solutions (1.3)**, provides comments from PwLC on benefits and suggested improvements to online delivery, “*I liked the format it was in because I could recap in my own time*” and “*Virtual works well—introduction would be good for members to have extra 15 min at the end*—*‘how is everybody?’ does anyone have any feedback to share... a bit of community spirit face to face would be difficult...who is in the group*”. A significant number of PwLC expressed interest in a face to face (in-person) delivery, with the perceived benefits of peer support being a consistent comment. This was moderated by the recognition of the logistical difficulties in achieving this (sub-themes **Face to face interactions 1.3.1** and **Session: Format/Style 1.3.2**). For example, “*A meeting in a room with everybody—especially now that covid is not so bad at present”, “I wonder if in an in-person group would be better—although whether people would do all 10 weeks?”,* and “*I think face to face for first and closing session again for that peer support side of things. Hybrid type session might work.*” Conversely however, a PwLC stated that “*Travelling and driving to a meeting would be too exhausting. Parking spots might be hard for people. I don’t know. I think probably doing it like that was good as some people joined in from work*” (PwLC).

#### 3.2.2. Main Theme 2: Content

In terms of main sub-theme **Acceptability (2.1)** and sub-theme **Just right (2.1.1),** the content was perceived to be “*Just right*” (PwLC), “*no, I feel it is very comprehensive, I was surprised by this*” (PwLC) and “*mostly the right sort of amount, felt there was no technical jargon and kept it straight forward, explained any medical terms used*” (PwLC). Some comments suggested that greater depth of information could have been provided, “*I could have done with a bit more on some stuff*” (PwLC) and “*For me a bit too basic*” (PwLC). 

Both groups had agreement within sub-theme **Unacceptable/unhelpful/lacking (2.1.3)**, specifically within unnecessary repetition during the VRP, for example: “*felt the repeated sessions weren’t needed, felt I was left searching for more information rather than repeated information, looking for ways to improve symptoms further but felt repeated from the first sessions. Felt that it was making me tired and wanting to save energy for other things so felt repeated information wasn’t the best use of my time*” (PwLC). The HCPs stated, “*Some elements repetitive through the course*” (HCP), “*Less duplication throughout the programme-better flow*” (HCP). 

Main theme **Value 2.4** and **Unique Value** sub-theme **2.4.1.1** represents comments from PwLC on the value of aspects of the VRP, for example, “*There were things I could pick up and I continue to do. It wasn’t overwhelming” “thank you for the course. I was desperate and not feeling well and not used to be on long term sick the course and team really helped me and really listened. it was really reassuring*” and “*I liked the format it was in because I could recap in my own time.*”

#### 3.2.3. Main Theme 3: Use of Digital Technology

Persons with LC describe the challenges and benefits of MT platform of the VRP within main Sub-theme **Experience (3.1)** including sub-theme **Platform (participation) (3.1.1)**, for example, “*struggled to participate due to use of MS teams—struggling to work out how to contribute to discussions*” (PwLC)*, “MS teams—liked being able to ask questions*” (PwLC) and “*could also send a message on group chat” (PwLC)*. Cognitive impacts of a virtual course were also expressed “*When you’re on Teams call it’s difficult to concentrate*” (PwLC). 

The Healthcare Professionals aims of the VRP in sub-theme **Delivery (3.3),** were: “To deliver intervention from multiple professions in a safe and condensed way” (HCP), “Provide therapy interventions to a larger group of people” (HCP) and “can reach a wide audience at once” (HCP). Further relevant HCP only Main Sub-themes and sub themes are represented in bold (Figure 2), including the need to re-design slides. 

#### 3.2.4. Main Theme 4: Group Dynamics

This theme includes main sub-themes around **Interaction (4.1)** and **Participation (4.2)***,* with **Positives (4.1.1)** that describe storytelling and sharing experience, for example: “*I thought that was good. Other people with similar problems*” (PwLC)*, “Overall yes. Big thing with long covid is the isolation—you realise someone else is verbalising what you are feeling—real value in that!*” (PwLC) and “*to have that discussion with a support network who had understanding and in the same position was really healing and reassuring*” (PwLC). However, there were drawbacks, as expressed in **Participation (4.2)** sub-theme **Barriers (4.2.2)** with comments such as “*I found very little of the end of session discussions to be relatable to my situation*” (PwLC), and “*I feel the peer support is important and I didn’t really get that from the session*” (PwLC). Some participants found that group size was an issue, solutions included*, “If groups too big use break out rooms, and rotate between*” (PwLC). The ability to have partners attend the VRP was also proposed, “*Perhaps partners being invited on it would be helpful. They are still learning...like with brain fog if your partners there they ask questions*” (PwLC). Within the sub-theme of **Benefits of Participation (4.2)**, PwLC reported that they no longer felt alone and shared the struggle, “*Because long covid thinking no one understood or going through it, sense of being in it together with others. Some useful tips...useful to hear*” (PwLC)*, “I did recognise that there were people worse than me. I was able to share my experience*” (PwLC) and “*Patients seem to like the Q&A/peer support at the end of each session*” (HCP). **Barriers to Participation (4.2.2)** co-existed, for example “*people and myself were reluctant to give information out due to embarrassment*” (PwLC) and “*This can be quite difficult for those who have fatigue*” (HCP).

#### 3.2.5. Main Theme 5: Individual Factors

For some with pre-existing long-term conditions there are implications for LC self-management strategies in main sub-theme **Pre-Existing Conditions (5.1)**, “*sessions were aimed at people who were well and then had long covid, whereas I feel I have already been through that process*” (PwLC). Participants described barriers to applying new skills and knowledge into practice “*I have to work, do my dissertation, everyday things I have to do, LC advice goes against my brittle asthma advice from my other doctors which can be a barrier*” (PwLC). Persons with LC describe fear of the return of LC symptoms and uncertainty, for example “*Some useful tools but worry that this will re-occur*” (PwLC). The sub-themes of **Individual care needs (5.3)** and, lack of **Adaptability (5.3.1)** was recognised by HCPs, they felt that the VRP was, “*not flexible or adaptive to individuals, some people will want more info quicker and others a slower pace*” (HCP), “*It doesn’t fit well as a one size fits all*” and “*having to cover all information to accommodate a wide audience—so some information may not be relevant to some patients*” (HCP).

#### 3.2.6. Main Theme 6: Internal Change

This final, substantial main theme includes sub-themes of **Reflection (6.1)** and **Positive reflection (6.1.1)** demonstrated by: “*absolutely, to understand what’s going on in my body and knowing how I can manage this is the key; diet was really useful*” (PwLC), “*It made me realise I was doing well compared to others on the call. I’m doing quite well actually*” and “*Yes I think it has given me things to help myself*” (PwLC). The sub-theme **Negative reflection (6.1.2)** is supported by examples such as “*Exs & Act: made me feel bad about myself—muscle loss made me panic about that...doesn’t mean people don’t need to know*” and “*it certainly helped, I feel I have a lot of problems due to covid that could not be helped by the virtual course” (PwLC)*.

Examples that underpin the themes of **Self-Efficacy (6.2)**, **Recognising self-efficacy (6.2.1)** and **Supporting self-efficacy (6.2.1.1)**: “*symptoms diary helped me understand what I can do and helped me build my activity;” “I suppose it has—it’s done things and helped with my own expectations. Once those are in place—I’m mentally in tune. There are always things you can try and give it go; you need to be open to the idea of getting better*” and “*sometimes you need that validation*” (PwLC). The themes of **Self-management (6.3)** and **Self-management learnings (6.3.1)** included **Pacing (6.3.1.1)** for PwLC: “*working within the boom-and-bust cycle ensured I could do the skills;” “I now meditate three times a day at work. Reduced workload at work. I have learnt to be a bit more selfish so that I am not too tired. I do breathing exercises. I monitor myself at work and I now have some energy outside of work. I feel that managing my energy better is down to the virtual course*” and “*Pacing myself. Plan more each day. Take each day as it comes*” (PwLC). Within Comments on **Breathing (6.3.1.2)**, “*Really good resources/detailed information... breathlessness one was really good, empowering, positive*” and “*Causes of Breathlessness. Re-gone over this and that has helped to use as self-correct (looked in mirror)*” (PwLC). Diet and nutrition were also important self-management learnings, “*absolutely, to understand what’s going on in my body and knowing how I can manage this is the key; diet was really useful*” and “*Some of the information in the ‘Diet’ session spurred further changes to my diet that have been beneficial*” (PwLC).

Within the theme of **Self-management leanings (6.3.1), Measuring improvement (6.3.1.4)** emerged, for example: “when I started the course I physically couldn’t get around, by the end of the course I had returned to work” and “75% yes I can manage more” (PwLC). This theme sits alongside the theme of **Goal setting (6.3.1.5)**, “Maybe not 100% but contributed—Having some personal goals in discussion with therapist and course would be helpful” (PwLC).

The sub-theme **Mechanisms for Knowledge gains (6.3.2)** was noted by both HCP and PwLC, for example: “I found more things out after finishing the course” (PwLC); “Sometimes I plan for a special occasion and then know I’ll need to rest for a few days. I avoid that on a day-to-day basis and have reduced my working hours to help with fatigue” (PwLC) and “how to apply what is being learnt” (HCP). Sub-theme **Advancing Self-management (6.3.3)** provides examples of the perceived benefits of combining rehabilitation approaches and delivery by HCPs only: “support the rest of the therapy input delivered in the service”; “allow 1:1 clinic time to be used for more individualised therapy” and “they are expected to try things out to then discuss at the reviews; it is not a passive course” (HCP).

## 4. Discussion

Overall, this service evaluation demonstrates that the VRP was well received and of value to PwLC who attended the sessions and took part in the evaluation. Importantly, it was able to inform changes to future VRP with a degree of clarity and collaboration, potentially limiting the risks of abandoning beneficial features. The authors recognize that there are some overlaps between the emergent themes, but are assured that all are valued and contributed to the development of the VRP. This work may represent a potential ‘blueprint’ for the development of Standards of Care in the delivery of an online or virtual intervention in LC. 

The main theme of **Attendance and Accessibility** outlined some of the valued aspects of the VRP and identified some of the barriers encountered. The ability to return to session slides was considered helpful. There was recognition that a digital delivery enabled the self-management of symptoms such as fatigue. Statements within sub-themes **Technology & links** and **Cognitive** highlight the potential barriers to a weekly online delivery of a VRP for PwLC with ‘brain fog’ (cognitive impairment). A quarter of PwLC who attended more than 50% of the sessions, identified Work/Life (50%) as more of a barrier than technical issues (20%) and Format/Content (20%) combined. The main sub-themes of **Environment** and **Work** illustrate that LC symptoms may be potentially under recognised and/or poorly understood by employers, with the allocation of time to attend a VRP during work hours being problematic. A similar issue was evident within the sub-theme **Home**, where attendance on the VRP may not be prioritised over other home demands. 

**Content** of the VRP was generally ‘just right’ however, unnecessary repetition in some sessions was unhelpful to PwLC and was identified as an issue by HCP. The presentation appearance and content were highly valued by PwLC allowing people to return to sessions in their own time and at their own pace. It is important to understand the significance of the non-linear pattern of symptoms and recovery in LC [28,29]. 

The **Use of Digital Technology** provided insights into the benefits and drawbacks of an e-health delivery that is now part of many health offerings, particularly for people with long term conditions [3]. Quantitative data showed that 42% of PwLC experienced difficulties with MT, 16% of these specifically with the MT digital link. Being new to technology and having difficulty with stable Wi-fi combined represented 26% of the total barriers expressed. This supports the presence of inequalities within both digital literacy and data poverty known to exist in the UK [3,29]. The COVID-19 pandemic has exposed many health and social inequalities, what remains unknown is how to address these and how best to improve access to digital health and self-management support for everyone [29].

The **Group Dynamics theme** sheds light on both the importance of sharing one’s experience and the isolating effects of LC. However, a digital delivery was not for everyone, some PwLC found it difficult to participate in group discussions: from a personal perspective; because they could not see who was in the group with computer cameras turned off and because of Wi-fi connectivity issues.

**Individual Factors** was the least obvious theme at the start of the TA. Long COVID can have a significant impact on people with pre-existing or multiple co-morbidities. The LC rehabilitation advice can be juxtaposed to that for pre-LC health management, therefore good health prior to a LC diagnosis should not be assumed. A ‘one size fits all’ approach may not allow for holistic management of PwLC and 1:1 support may also be required for tailored support.

The final theme of **Internal Change** provides an early footprint as to how PwLC may learn to self-manage their complex condition. Sub-themes of **Self-efficacy**, **Self-knowledge** and **Measuring improvement** at an individual level suggest that standardised LC patient reported outcome measures, such as condition specific C19-YRS [19,26,30], may have utility for this cohort and aid self-management support. More research in this area is needed.

Gathering quantitative and qualitative data reveals convergence points on **Attendance and Accessibility** and related sub-themes. There is clear agreement between sessions most attended and those deemed most useful by PwLC (Figure 1). ‘Session 1: What is LC’ displays high value, as do sessions on fatigue and breathing. However, there was divergence between data with regard to diet. Although, more than 50% attended this session there is almost equal divide between ‘Most useful’ and ‘Least useful’ session (Figure 1). Comments suggest a latent value, with PwLC expressing benefit when they re-accessed the presentation. Perhaps the more impactful symptoms of fatigue and breathlessness inhibit thoughts or actions on diet, indicating that the individual timeframe and unpredictability of LC is significant, a reminder that a ‘one size’ fits all intervention is less helpful [29,31]. 

Healthcare Professionals may be more aware of other socio-economic barriers to digital health delivery, derived from clinic interactions and comments such as: “*who can afford the device*” and “*have the IT skills and be time rich.*” One PwLC, with no IT knowledge, did not attend any sessions or receive slides by email. This is a concern and may be representative of other PwLC who did not respond to the invitation to participate [31]. Although it is accepted that some PwLC struggled to concentrate secondary to LC symptoms such as ‘brain fog’ (cognitive impairment) and fatigue, the VRP allowed for the delivery of LC education, support management and much appreciated peer support to a greater number of people than would be possible in an in person setting. This model of care appears compatible with availability of funding and qualified members of the MDT, holds value and is therefore likely to be a sustainable option in an overburdened NHS service. 

The HCP delivering the VRP had to focus on delivering timely, self-management support to the greatest number of people in the presence of long waiting times, and in the knowledge of significant delays in PwLC being diagnosed and referred into the service. Although the PwLC demographics of this evaluation are in keeping with the received referrals, it is not representative of the wider population suspected to have LC within the City, indicating that more work on reaching these less well represented communities is important.

### 4.1. Impact of the VRP Evaluation

The findings of this service evaluation underpinned the development of the VRP, and gave clear direction for change to the service delivery team. For example:

- At the start of ‘Session 1’, five to ten minutes are spent familiarizing participants with ‘Microsoft Teams’ software
- A section about pain has been added and the mental well-being session condensed. All presentations are now capped at 30 min to support engagement and allow extra time for peer reflection.
- A two minute ‘pause’ in each session re-enforces advice given from the HCPs about taking a break from activity, regular movement and hydration.
- Sessions 3 and 9 (Causes of Breathlessness and Breathing Retraining) are now more practical and interactive, this has resulted in better discussions between participants at the end of the sessions
- Video recordings for each session are now sent to participants instead of Microsoft PowerPoint presentations so they can re-watch at times that suit.

### 4.2. Strengths

This paper is important for patients, it is the first to describe and evaluate VRPs for PwLC. A search of the Clinical Trials Register and evidence databases failed to identify any similarly designed VRP for PwLC studies at this time. However, a recent website post of an MDT VRP based at The Ottawa Hospital (Canada) [32] has received good reviews, with 40 PwLC accessing their programme to date. 

This work was conducted by two CRFs embedded within a clinical MDT, enabling scientific evaluation of the impact of clinical interventions. This service evaluation was developed in collaboration with both the clinical and research team to ensure that the information collected was the most useful for informing future practice. Whitehouse et al. [33] suggests that this partnership between HCPs and academics is incredibly valuable and creates the ability to create capacity and capability within health care services.

Having funded CRFs embedded within a clinical team is a new model for Health Services, with major impact on evidence-based practice and collaboration with people who use health care services [33,34]. Benefits to individual CRFs include academic supervision and career progression.

A mixed method design strengthened this service evaluation of a digitally delivered, complex intervention for PwLC. Little is known still about LC, in addition to the benefits and drawbacks of e-health delivery. The use of both quantitative and qualitative data allowed for a good level of triangulation, identifying areas of successful practice, aspects requiring no additional change, and where changes/improvements could be made.

### 4.3. Limitations

This service evaluation has a few limitations. It highlights the views of PwLC but was not co-produced, which could have enhanced the depth of analysis. The authors did not explore the reasons behind why some PwLC did not attend the VRP. Partners, family, carers, or other significant people to PwLC were not included in this service evaluation as they were not invited to attend the VRP. They may have been able to provide additional insight on the impact the VRP on PwLC and potential improvements.

Although the CRF are non-clinical members of the MDT/service they may have been perceived by PwLC as being part of the clinical team. There may have been a bias to report positively on the service, equally PwLC may have not have fully disclosed their views due to concern around on-going care in the service. This was mitigated against by clear identification and communication of their role in the service with open and honest feedback encouraged to improve service quality.

The demographics of the PwLC involved in this service evaluation were representative of the population referred to the Leeds LC Rehabilitation Service. The demographics of those who participated may not be representative of the population that is predicted to have LC, such as those in diverse and less well represented communities.

There is a recognised need to co-design services and co-produce research as a component of best practice. To address this, a Patient Carer Public Involvement (PCPI) Group is under development. The team hopes that this will support comprehensive co-production of services, ongoing evaluation, and research development.

### 4.4. Recommendations

The authors suggest that the results of the VRP evaluation could be considered as a guide for other MDTs during the design of VRPs for PwLC (see Table 3).

In summary, the authors recommend that a VRP is co-produced with PwLC, carers, members of the public with an interest in LC and those with long term conditions, to ensure that it meets the needs of users within the community.

The authors recommend that further research be carried out to: (a) understand how VRPs could be accessed in communities that have challenges within digital literacy and data poverty, (b) evaluate the impact of delivering a VRP in terms of cost analysis and patient outcomes.

## 5. Conclusions

The authors found that the VRP was highly regarded by PwLC who attended the course, particularly the sessions around key symptoms such as breathlessness and fatigue. The opportunity for sharing stories and experiences within the peer discussion was also valued.

Our results are available to support the development of LC VRPs.

The authors suggest that the themes and recommendations described in Table 3 and could be applied to the development of VRP within other clinical fields.

## Figures and Tables

**Table 1:** Sessions from Virtual Course for Symptom Management of Long COVID.

Session	Topic	Lead Facilitator
1	Understanding LC	Medical Doctor, Occupational Therapist, Physiotherapist (Clinical co-ordinators)
2	Fatigue Management Part 1	Occupational Therapist
3	Causes of Breathlessness	Physiotherapist
4	Sleep and Relaxation	Occupational Therapist
5	Mental Wellbeing	CBT Therapist, Occupational Therapist.
6	Diet	Dietician
7	Exercises and Activity	Physiotherapist
8	Fatigue Management Part 2	Occupational Therapist
9	Breathing Retraining	Physiotherapist
10	Progressing Exercise and Activity	Physiotherapist

**Table 2:** Persons with Long COVID (PwLC) demographics.

Demographic Data	N = 38
Gender	F 26 (68.42%)
	M 12 (31.57%)
Age (years)	25–34 5 (16%)
	35–44 7 (19%)
	45–54 9 (22%)
	55–64 8 (19%)
	65–74 6 (13%)
	Not Known 3 (11%)
Ethnicity	White: 31 (78.94%)
	Asian Indian: 3 (7.89%)
	Asian Pakistani: 2 (5.26%)
	Black African: 1 (2.63%)
	Mixed White and Black Caribbean: 1 (2.63%)
Duration of LC symptoms	3–6 months 0
	6–12 months 0
	12–18months 20
	18–24 months 18

**Table 3:** Recommendations for the design of Virtual Rehabilitation Programme in Long COVID.

Theme	Recommendation
1. Attendance and Accessibility	Deliver sessions at a time that minimises burden of symptoms and consider number of sessions Engage with workplace and enable attendance for PwLC who are working Consider inclusion of “in person” elements where possible Utilise the skills and knowledge of the MDT to address the multi system demand of LC
2. Content	Combine MDT knowledge and current evidence base when designing VRP content Limit length of ‘taught’ programme content to between 20 and 30 min Focus on key symptoms specific to population requirements Enable post session access to VRP content Included welfare rights Include information about current research
3. Use of Digital Technology	Use a free to use video conferencing platform Provide IT support and access Monitor and include chat function within the group
4. Group Dynamics	Ensure introductions at the start Set “ground rules” for use of webcams for participants and facilitators Use break-out rooms for peer discussion in larger groups Provide opportunity and facilitation for peer discussion
5. Individual Dynamics	Consider accessibility for those with specific needs such as: hearing and visual impairment, those with language barriers and those from less well represented communities Invite partners, carers, or those of importance to programme participants
6. Self-management	Include elements of interaction such as quizzes, self-reflection, practical exercises Include practical skills for self-management such as breathing techniques, fatigue management Acknowledge differing life situations such as: being parents, other caring responsibilities, employed/unemployed, age, menopause Include information on relapse of symptoms

## Data Availability

Anonymised data can be obtained by contacting the corresponding author.

## Supplementary Materials

The following supporting information can be downloaded at: https://www.mdpi.com/article/10.3390/ijerph191912680/s1, Figure S1: Healthcare Professional (HCP) Questionnaire; Figure S2: Person with Long COVID (PwLC) Questionnaire; Table S1: Combined Healthcare Professional (HCP) and Person with Long COVID (PwLC) Template Analysis with qualitative comments.
