# Supplementary material for: A Mixed-Methods Evaluation of a Virtual Rehabilitation Program for Self-Management in Post-COVID-19 Syndrome (Long COVID)
Source: Int J Environ Res Public Health. 2022 Oct 4;19(19):12680. doi: 10.3390/ijerph191912680 (PMC9566175; doi:10.3390/ijerph191912680)
Supplement: Supplementary file 1 [file ijerph-19-12680-s001.zip › Figure S1 Healthcare Professionals (HCP) questionnaire.pdf]

Figure S1: Healthcare Professional (HCP) Questionnaire

The survey will take approximately 4 minutes to complete.

As part of an ongoing review of the virtual course we would like to take this opportunity to ask you a few questions. This is entirely voluntary with all comments remaining anonymous. It is hoped the results may help identify themes that can be used to inform a service user questionnaire as part of a service evaluation. This will be used to make relevant changes to the course.

Thank you for your time,

Thuvia & Hannah, Clinical Research Fellows, Long COVID Rehabilitation Team

1.What is your profession?

2.How long have you worked in the Long Covid Rehabilitation Team?

3.What would you say are the key aims of the Long Covid Virtual course?

4.What do you feel are it's current strengths?

5.What do you feel are it's current limitations?

6.In your own words, can you name 3 things you perceive would improve the course and rank order them in terms of their importance? (*1. being most important, 2. next and then 3.)*

7.Please add any further comments relating to your choices for question 6 or any further points or suggestions you wish to make

Submit
