# Supplementary material for: A Mixed-Methods Evaluation of a Virtual Rehabilitation Program for Self-Management in Post-COVID-19 Syndrome (Long COVID)
Source: Int J Environ Res Public Health. 2022 Oct 4;19(19):12680. doi: 10.3390/ijerph191912680 (PMC9566175; doi:10.3390/ijerph191912680)
Supplement: Supplementary file 1 [file ijerph-19-12680-s001.zip › Figure S2 Person with Long COVID (PwLC) questionnaire.pdf]

Figure S2: Person with Long COVID questionnaire

Leeds Community Healthcare
NHS Trust

Leeds Long COVID Rehabilitation Virtual Course Evaluation

Business Unit: Specialist

Service: COVID-19 Rehabilitation Service

Service Team: COVID-19 Rehabilitation Service – Citywide

1: Do you consent to participate in this service evaluation?

- ☐ Yes
- ☐ No

2: Which sessions did you attend? (Please tick all that apply)

- ☐ Understanding Long Covid
- ☐ Fatigue Management
- ☐ Causes of Breathlessness
- ☐ Sleep and Relaxation
- ☐ Mental Wellbeing
- ☐ Diet
- ☐ Exercises and Activity
- ☐ Fatigue - Part Two
- ☐ Breathing Retraining
- ☐ Progressing Exercise and Activity

Leeds Community Healthcare NHS Trust

3: Which session(s) did you find most useful? (Please tick all that apply)

- ☐ Understanding Long Covid
- ☐ Fatigue Management
- ☐ Causes of Breathlessness
- ☐ Sleep and Relaxation
- ☐ Mental Wellbeing
- ☐ Diet
- ☐ Exercises and Activity
- ☐ Fatigue - Part Two
- ☐ Breathing Retraining
- ☐ Progressing Exercise and Activity

4: Please tell us why you found these sessions useful?

☐ Please tick this box if you do not wish your comment to be made public.

Leeds Community Healthcare NHS Trust

5: Which session(s) did you find least useful? (Please tick all that apply).

- ☐ Understanding Long Covid
- ☐ Fatigue Management
- ☐ Causes of Breathlessness
- ☐ Sleep and Relaxation
- ☐ Mental Wellbeing
- ☐ Diet
- ☐ Exercises and Activity
- ☐ Fatigue - Part Two
- ☐ Breathing Retraining
- ☐ Progressing Exercise and Activity
- ☐ Not Applicable

6: Please provide comments.

☐ Please tick this box if you do not wish your comment to be made public.

Leeds Community Healthcare NHS Trust

7: If you attended half or less of the sessions, what were the reasons for this? (Please tick all that apply)

- [ ] Format and Content
- [ ] Work/Life Demands
- [ ] Symptoms or Illness
- [ ] Course was no longer required
- [ ] Other, please specify:

Other, please specify:

8: Overall, what do you think about the amount of information given in the sessions?

- [ ] Too Much
- [ ] Just Right
- [ ] Too Basic

9: Please provide any comments.

- [ ] Please tick this box if you do not wish your comment to be made public.

10: Did you find the closing discussions at the end of each session useful?

- [ ] Yes
- [ ] No

NHS
Leeds Community Healthcare
NHS Trust

11: Please provide any comments.

☐ Please tick this box if you do not wish your comment to be made public.

12: What do you think about the group size?

☐ Too Big

☐ Just Right

☐ Too Small

13: Please give comments.

☐ Please tick this box if you do not wish your comment to be made public.

14: Were you able to put into practice any of the skills that were recommended in the course? (For example, planning, pacing and prioritising, boom and bust cycle, activity diaries, breathing exercises).

☐ Yes

☐ No

NHS
Leeds Community Healthcare
NHS Trust

15: Please tell us more about the skills you were you able to put practice?

☐ Please tick this box if you do not wish your comment to be made public.

16: Were there any barriers to completing this? What were they?

☐ Please tick this box if you do not wish your comment to be made public.

17: Have you used Microsoft Teams (online video calling) before?

☐ Yes

☐ No

18: Did you have any problems using Microsoft Teams whilst attending the course?

☐ Please tick this box if you do not wish your comment to be made public.

Leeds Community Healthcare NHS Trust

19: If the course was to be delivered using another format, what would you like it to be?

☐ Please tick this box if you do not wish your comment to be made public.

20: Were the times and days of the course convenient for you?

☐ Yes

☐ No

☐ Not Applicable

21: If no, which days would be most convenient for you? (Please tick all that apply).

☐ Monday

☐ Tuesday

☐ Wednesday

☐ Thursday

☐ Friday

☐ Saturday

☐ Sunday

☐ Any Day

Leeds Community Healthcare NHS Trust

22: Please tell us what time would be best for you?

- [ ] 8am - 10am
- [ ] 10am - 12pm
- [ ] 12pm - 2pm
- [ ] 2pm - 4pm
- [ ] Evening
- [ ] Any time

23: Overall, do you feel the course has provided you with the knowledge and skills to manage your Long COVID?

- [ ] Yes
- [ ] No

24: Please provide any comments.

- [ ] Please tick this box if you do not wish your comment to be made public.

25: Was there anything else you felt could be included in the course?

- [ ] Please tick this box if you do not wish your comment to be made public.

26: Please use this box to leave any other feedback or comments you may have.

☐ Please tick this box if you do not wish your comment to be made public.

About you

27: What age are you?

☐ 16-24

☐ 25-34

☐ 35-44

☐ 45-54

☐ 55-64

☐ 65-74

☐ 75-84

☐ 85+

28: What is your ethnic group?

- ☐ White - English/Welsh/Scottish/Northern Irish/British
- ☐ White - Irish
- ☐ White - Gypsy or Irish Traveller
- ☐ Other - White
- ☐ Black - Caribbean
- ☐ Black - African
- ☐ Other - Black
- ☐ Mixed - White and Black Carribbean
- ☐ Mixed - White and Black African
- ☐ Mixed - White and Asian
- ☐ Other Mixed
- ☐ Asian - Indian
- ☐ Asian - Pakistani
- ☐ Asian - Bangledeshi
- ☐ Asian - Chinese
- ☐ Other Asian
- ☐ Other - Arab

Other, please specify:

Leeds Community Healthcare NHS Trust

29: Are you;

- ☐ Male
- ☐ Female
- ☐ Transmale
- ☐ Transfemale
- ☐ Non-binary
- ☐ Do not wish to say

30: Please tell us your occupation.

Please return this survey to the patient experience team.
