# Supplementary material for: A Mixed-Methods Evaluation of a Virtual Rehabilitation Program for Self-Management in Post-COVID-19 Syndrome (Long COVID)
Source: Int J Environ Res Public Health. 2022 Oct 4;19(19):12680. doi: 10.3390/ijerph191912680 (PMC9566175; doi:10.3390/ijerph191912680)
Supplement: Supplementary file 1 [file ijerph-19-12680-s001.zip › Table S1 Combined HCO and PwLC Template Analysis with qualitative comments.pdf]

Table S1: Combined HCP and PwLC Template Analysis with qualitative comments

‘Main Themes	Main Sub-Themes	Sub Themes	
**1. Attendance & Accessibility**	**1.1 Barriers**	**1.1.1 Technology & link**<br>• technology issues (C101, Q4)<br>• had issues logging on initially when enrolled onto two courses, now enrolled onto a third course and IT issues have resolved (C101, Q10).<br>• had to ring up several times for the link as hadn't received it. struggled to use it in general (C103, Q10).<br>• Difficulty with link so couldn’t join (C113, Q4).<br>• *Links can be more cognitively challenging need to have value indicated (C232, Q15).*<br>• A chance for those who can afford the device, have the IT skills and are time rich to learn more to aid their rehabilitation (S11, Q4).<br>• Those who have economic, time, IT education or language disadvantages can’t access. Commitment to 10 sessions over 10 weeks is a lot to commit to. There is no option for attending course at a site with help / support / IT provision (S11, Q5).<br>• Access - for those clients who are reluctant to attend or there are IT barriers (S16, Q6).	
		**1.1.2 Environment**	**1.1.2.1 Work**<br>• work got in the way (C111, Q8b).<br>• Fitting into work time (C116, Q4).<br>• Work/Life demands (C215, Q4)<br>• Tricky as I don't think I committed to it due to work (C232, Q2a).<br>• Full time employment. (C2 116, 8b)
			**1.1.2.2 Home**<br>• I think its challenging even being at home (C109, Q8b).<br>• Life - home circumstances (C226, Q8b).<br>• home life (C2 10 Q8b)<br>• It was unable to attend any of the others due home circumstances (C2 26, Q2a).<br>• I didn't get to do the course or have time to look at slides so no. (C226, Q8a)<br>• Life - home circumstances. (C226, Q8b).
		**1.1.3 Cognitive**	

		• At first I didn't or wasn't able to absorb information at time. Yes as I didn't really know what I was doing (C105, Q10).<br>• peoples understanding of what long covid is (C113, Q8b).<br>• recording the activity diary was a bit of a chore sometimes due to demands of my energy to complete it. (C224, Q8b)<br>• I think the 9.30am a bit early. 10 or 10.30 (C113, Q3a).<br>• When your on Teams call it’s difficult to concentrate (C114, Q3a).<br>• some patients may find it frustrating having to wait so long to get all the information (S3, Q5).	
		**1.1.4**	
		**1.1.4 Accessibility**	**1.1.4.1 Language Barriers**<br>• Not accessible for patients who have language barriers, learning disabilities or hearing impairments (S10, Q5).<br>• More accessible to all patients dependent on language, disability, impairments (S10, Q6).
			**1.1.4.2 Health**<br>• Some who are more unwell and sometimes juggling many appointments or bad days with the course (S4, Q5).<br>• health inequalities (S10, Q6).<br>• found that progressing exercises and activity was difficult as couldn't exercise more (C103, Q3a).
	**1.2 Enablers (Compatibility with main symptoms)**<br>• liked being able to read the slides after (C103, Q11).<br>• Never quite full hour there was conversation in between. If it had a full hour, I might not have coped (C105, Q5a).<br>• Travelling and driving to a meeting would be too exhausting. Parking spots might be hard for people. I don't know. I think probably doing it like that was good as some people joined in from work (C105, Q11).		

	• ms teams - liked being able to ask questions (C111, Q11).<br>• I liked the format it was in because I could recap in my own time (C226, Q11).<br>• Virtual works well - introduction would be good for members to extra 15 mins at the end - 'how is everybody?' does anyone have any feedback to share... a bit of community spirit Face to face would be difficult...who is in the group. Mixed group would be challenging (C232, Q11).<br>• Virtual session better attendance - odd occasional in person sessions - drop in or option Overall virtual best (C227, Q11).<br>• sessions on Mondays and Fridays to further increase the times available for patients to attend (S2, Q6).<br>• Flexible agreements in terms of patients accessing the course as above (S4, Q6).<br>• Making it more accessible to those who can't attend on the day (S7, Q6).<br>• Offer alternative or those who struggle to attend at a set time (S8, Q6).		
	**1.3 Options/solutions**	**1.3.1 Face to face interactions**	**1.3.1.1 Improved understanding/depth**<br>• I feel face to face works a lot better. (C113, Q11).<br>• possibly to go more in depth than the course to follow up the live session (C113, Q11).<br>• face to face. i'm not very good with computers. (C218, Q11).<br>• Maybe 2 meet ups in-person: 1 at the beginning and then near the end. The rest virtual (C228, Q11).<br>• face to face (C225, Q11).
			**1.3.1.2 Peer support & sense of group**

			• A meeting in a room with everybody - especially now that covid is not so bad at present (C114, Q11).<br>• I think face to face group. It would be like...for it to sink in and be able to speak and see people (C116, Q11).<br>• I wonder if in an in-person group would be better - although whether people would do all 10 weeks? (C213, Q6a).<br>• People may contact one another and maybe meet up (C114, Q11).<br>• I just think everybody meeting up would be the best. I think people would make the effort to go (C114, Q15).<br>• I think face to face for first and closing session again for that peer support side of things. Hybrid type session might work (C216, Q11).<br>• Coffee group feeling might be better (C232, Q6a).<br>• Maybe a class I would be ok with (C229, Q11).
		**1.3.2 Session: Format & Style**	**1.3.2.1 Length of session**<br>• 1 hour was a bit long for me (C105, Q3a).<br>• 20 mins was long enough (C116, Q5a).<br>• feel the course sessions could be a little bit longer (C113, Q5a).<br>• The presentation can be quite long and the length ranges each session (S1, Q5).<br>• patients having to concentrate for long periods of time without actively participating or moving (S2, Q5).<br>• Some of the sessions can be longer in duration (S12, Q5).<br>• Sessions to run for the same duration (S12, Q6).<br>• The presentation can be quite long and the length ranges each session (S1, Q5).<br>• patients having to concentrate for long periods of time without actively participating or moving (S2, Q5).<br>• Some of the sessions can be longer in duration (S12, Q5).<br>• Sessions to run for the same duration (S12, Q6).
			**1.3.2.2 Times of session**

			• Offer a weekend/evening virtual or in person ? COVID drop in or regular session (C215, Q11).<br>• That later in morning was better 10 or 10.30am for times. Days of week were not a problem (C114, Q12b).<br>• Timings are not ideal for patients who are working (S2, Q5).<br>• 10 weeks is a large commitment (S3, Q5).<br>• others who are functional well but trying to juggling work etc again by then (S4, Q5).<br>• Limited flexibility with patients accessing course (set date/time) (S9, Q5).<br>• Not accessible for patients are don't have access to IT (S10, Q5).<br>• dates and times – access (S14, Q5).
			• **Mixed format**<br>• I enjoyed it online because it didn't take energy to get to a venue which enabled it to not affect my whole day. the recorded copy was really useful to share with family members (C234, Q11).<br>• Having the presentation and then a live peer discussion works well (S1, Q4).<br>• Having both information giving, and discussion also with others with Long COVID (S3, Q4).<br>• the mix of education and opportunity for questions/discussion is good (S17, Q4).<br>• Providing an option to attend something somewhere with support, as a group. Tie in with genuine peer support offer (S11, Q6).<br>• I think if there had been an open session in one of the weeks - so people could have an open discussion. we could chat and hcp could say yes that is a symptom, advise see gp, signpost (C213, Q11).<br>• It would be nice to have a follow up, or a drop-in session every week after the course. maybe with a theme for each week (C113, Q13a).
**Themes (A-Priori)**	**Main Sub-Themes**	**Sub Themes**	
2 Content	**2.1 Acceptability**	**2.1.1 Just right**<br>• Just right (C105, Q5a).<br>• They were ALL useful (C109, Q2a).<br>• I thought it was ok (C113, Q5a).	

		• it was perfect C225, Q5a).<br>• no - thank you for the course. I was desperate and not feeling well and not used to be on long term sick the course and team really helped me and really listened. it was really reassuring (C234, Q15).<br>• no, I feel it is very comprehensive, I was surprised by this (C101, Q14).<br>• mostly the right sort of amount, felt there was no technical jargon and kept it straight forward. explained any medical terms used (C113, Q5a).	
		**2.1.2 Too little/basic**<br>• I could have done with a bit more on some stuff (C109, Q5a).<br>• some felt too basic, and some felt ok (C111, Q5a).<br>• For me a bit too basic (C117, Q5a).<br>• I don't think it went into depth enough - didn't explain enough. Didn't emphasis that's not the virus its how your body reacts to it. It left a footprint (C226, Q5a).<br>• Overall I had a lot of knowledge but understand why it was basic, but other bits like fatigue I knew less about (C215, Q5a).<br>• All very relevant. Only what I will say is - 10) wasn't applicable to you at that as I was just able to walk. The more intensive items (C214, Q3a).<br>• Session 10: It adds to the feeling you should have improved by now (C228, Q3a).<br>• Diet : such an individual thing. Quite entry level and I'd done a lot of trial and error (C117, Q3a).	
		**2.1.3 Unacceptable/unhelpful/lacking**<br>• something about injuries caused to the body by long covid e.g., kidneys, liver, heart or questions like "will my lungs be permanently damaged?" (C211, Q14)<br>• Providing recommendations of books: Cured. About your mindset and this can aid recovery. Being positive. Hope (C214, Q14).<br>• I think anything to do with legal side of work. What your rights were if you need time off or to reduce your hours (C216, Q14).<br>• 'Administration of my illness' – it’s like a project. Benefits & Finance: how to manage or how to manage appointments - sources of help for benefits. This has affected my mental health (C228, Q14).	

		• breathing. a video of what to do when you actually have covid e.g breathing exercises as I caught covid for a second time. or some advice of what can help with active covid (C113, Q13).<br>• No discussion on pain, brain fog, return to work and benefits (S5, Q5).<br>• If the rehabilitation information / skills need to be given to patients there needs to be other options for giving it to those struggling to keep their jobs etc (S11, Q5).
	**2.2 Coherence**<br>• Some I thought same thing was being repeated Others just right (C213, Q3a).<br>• some of the sessions repeated themselves, felt these didn't need to be there (C236, Q3a).<br>• felt the repeated sessions weren't needed, felt I was left searching for more information rather than repeated information, looking for ways to improve symptoms further but felt repeated from the first sessions. Felt that it was making me tired and wanting to save energy for other things so felt repeated information wasn't the best use of my time (C234, Q3a).<br>• some duplication of information throughout the course content (S1, Q5).<br>• Some elements repetitive through the course (S8, Q5).<br>• Less duplication throughout the programme- better flow (S1, Q6).<br>• Less repetitive content (S7 Q6).	
	**2.3 Utility**	**2.3.1 Written**<br>• finds it useful to makes notes of the hard copies. finds the hard copies really useful (C101, Q5a)<br>• very good information (C103, Q5a).<br>• was bit scared at first and anxious by the postural hypo tension. Once I went back over the slides I realised it was part of it - but I didn't have it (C105, Q2a).

		• No I think they covered most of the things you need (C105, Q14).<br>• one the slides mentioned how long long covid lasts for, I feel my long covid has lasted longer than the longest time scale mentioned on the slides (C111, Q6a).<br>• the slides helped me learn the breathing techniques. it helped me learn a completely new way of breathing (C218, Q13a).	
		**2.3.2 Increased understanding**	**2.3.2.1 Condition**<br>• how to best manage symptoms (S9, Q3).<br>• Providing Information to aid patients manage their symptoms (S11, Q3).<br>• giving people information to understand their experience better and self-manage symptoms (S4, Q3).<br>• to reduce impact of Long Covid (S13, Q3).
			**2.3.2.2 NHS services**<br>• surprised with demands on resources that the course was available (C101, Q15).<br>• Took a while to get an appointment. The 1st time I went I felt people understood me and had dealt with people who had symptoms. Being honest and saying didn't came away feeling like someone did understand (C213, Q15).
		**2.3.3 Timing personal**	
	**2.4 Value**	**2.4.1 Overall value**	**2.4.1.1 Unique value**<br>• mental wellbeing - felt this could have been longer (C113, Q3a).<br>• Progression Ex & Act: didn't fit me at this time (C117, Q3a).<br>• There were things I could pick up and I continue to do. It wasn't overwhelming (C228, Q5a).<br>• Fatigue diary/ 3 P/ Breathing exs - sat well with Dr Corrado's study (C228, Q8a).<br>• **VC overall really good, useful, opportunity to liaise with other LC team in general gave me hope when I felt most helpless. GP - wasn't listening. really supported service. Thank you ! (C2 27, Q15).**<br>• felt it ticked most boxes (C2 36, Q13a).

			• **no - thank you for the course. I was desperate and not feeling well and not used to be on long term sick the course and team really helped me and really listened. it was really reassuring (C2 34, Q15).**<br>• I liked the format it was in because I could recap in my own time. (C226, Q11).
			**2.4.1.2 Practical value**<br>• ideas: Breathing at the start of every session to remind people how important it is. (Charlie mentioned it). I said to Charlie someone like me session one - a headline of each session would be good or suggested ideas. A snapshot: Med diet/ breathing ex's People could then go away and start reading or looking at it.<br>• varied a lot between sessions, the early sessions were heavy with important science, felt this could have been spread out a bit more in smaller chunks throughout the course (C234, Q5a).<br>• Useful information (S4, Q4).<br>• Lots of information (S8, Q4).<br>• Pitched at a good level for most people to understand but also gain information (S9, Q4).<br>• The educational content (S10, Q4).<br>• they appreciate the educational content (S16, Q4).
			**2.4.1.1 Range of Topics**<br>• I didn't do all the courses but perhaps something about women's health (peri-menopause) (C232, Q14).<br>• covering a range of topics (S1, Q4).<br>• Wide range of topics covered (S2, Q4).<br>• Topics covered (S3, Q4).<br>• Informative Concise Broad spectrum of topics covered (S12, Q4).
			**2.4.1.2 Evidence Based**<br>• Very informative and evidence based, best practice (S3, Q4).<br>• Updated information which includes some background on the emerging evidence base (S1, Q6).<br>• updated content (S14, Q6).
		**2.4.2 Enhancing value (Knowledge needs)**	**2.4.2.1 Workplace, Welfare & Benefits**

			• dealing with workplaces that aren't helpful, mine haven't implemented the occupational health recommendations. having someone available to offer advise near the beginning of the course or contacts for advice (C111, Q14).
			**2.4.2.2 Visual resources**
			**2.4.2.3 Research needs & understanding**<br>• For me what would have been helpful would be to have the recordings. Especially for missed session Other thing was to summarise what the research is about To have a newsletter updating people on service and research. I'd be happy to be involved with that. (C216, Q15).<br>• *it would be useful to know up and coming research and to share things that are helping like alternative treatments. (C234, Q14).<br>• At end of course research update Long Covid, feels time sensitive people with Long Covid like to know they are being talk about. medical, pharmacology (C232, Q15).<br>• it would be useful to know up and coming research (C234, Q14).
			**2.4.2.4 Role for Social Media**<br>• Suzie Bolt Facebook group (C214, Q13a)<br>• Facebook group has been really helpful for my recovery. I think signposting people once they have been referred and are waiting to be seen by the service would be good (C214, Q15).
		**2.4.3 New horizons**	
	**2.5 Purpose**	**2.5.1 Education**	**2.5.1.1 Knowledge**<br>• plus an overview of Long COVID and understanding of what it does to the body (S10, Q3).<br>• education on the theories of why symptoms present (S7, Q3).<br>• To improve a patient's understanding of Long COVID and potential causes for their ongoing symptoms (S8, Q3).
			**2.2.5.2 Skills**<br>• Educate patients about how to best manage symptoms (S8, Q3).

			• Good overall overview of Long Covid and strategies (S15, Q4).<br>• More information about dealing with setbacks/relapses (S15, Q6).
			**2.2.5.3 Outcomes**<br>• To raise awareness of common symptoms following Long Covid (S12, Q3).<br>• To raise awareness and support with educating patients around associated common symptoms related to long covid (S11, Q3).<br>• To raise awareness and support with educating patients around associated common symptoms related to long covid (S10, Q3).<br>• To deliver typical information about common symptoms (S4, Q3).<br>• Good information about management of common symptoms (S9, Q4).<br>• To give an overview of the main symptoms of Long Covid (S15, Q3).<br>• To learn and understand Long Covid from an intellectual and emotional perspective (S5, Q3).<br>• Further education and background of Long COVID (S1, Q3).<br>• Educate and inform of the condition (S3, Q3).<br>• Educate patients (S9, Q3).<br>• Educate and provide self-management skills (S14, Q3).<br>• Educational programme (S16, Q3).<br>• Education (S17, Q3).
			**2.2.5.4 Alongside 1:1 Rehabilitation**<br>• demonstrate rehab technique (S6, Q3).<br>• rehabilitation of patients with long covid (S10, Q3).<br>• inclusive of rehabilitation techniques (S12, Q3).<br>• Regular recognition of need/value for therapy to live with Long covid (S5, Q4).
		**2.5.2 Delivery**<br>• Content Delivery method (S14, Q4).<br>• To deliver educational sessions to patients (S12, Q3).	
	**2.6 Engagement**	**2.6.1 Interactivity**<br>• Some lacked a bit of content with talking - then sometimes it would be better (C109, Q3a).	

		• Interaction would have been useful. SLIDES only (C2 15, Q13a).<br>• Teaching method is a bit dry and not as engaging as could be (S5, Q5).<br>• how interactive it is (S6, Q5).<br>• Not particularly interactive to watch so can get disengaged (S8, Q5).<br>• Slides are not as interactive ? reducing engagement (S9, Q5).<br>• Not interactive enough (S2, Q5).<br>• Limited ability to engage with patients on a face to face or practical basis (S9, Q5).<br>• I'm not sure about the voiceover element which seems a little impersonal, but would be a lot more intensive for staff to deliver talking through the slides in real time (S16, Q5).<br>• Less static presentation style - person talking in view/more practical (S5, Q6).<br>• be more interactive (S6, Q6).<br>• Encourage patient engagement through more interactive content (S7, Q6).<br>• More interactive (S7, Q6).<br>• Improve engagement with slides by breaking up presentation with practical or interactive element (S9, Q6).<br>• More practical work (S10, Q6).<br>• Practical element incorporated into more sessions (S12, Q6).<br>• Lack of practical elements (S12, Q5).<br>• No voiceover which allows for more interaction (S16, Q6).<br>• have some live teaching, eg. exercises, breathing, relaxation (S17, Q6).	
		**2.6.1 Continuity**<br>• Consistent thread throughout (S4, Q6).<br>• to improve content / order to ensure flows from one week to the next (S6, Q6).<br>• Continuity of therapists delivering the sessions (S13, Q6).	
**Themes (A-Priori)**	**Main Sub-Themes**	**Sub Themes**	
	**3.1 Experience**	**3.1.1 Platform (participation)**	

**3 Use of Digital Technology**		• struggled to participate due to use of MS teams - struggling to work out how to contribute to discussions.(C103, Q6a).<br>• ms teams - liked being able to ask questions (C111, Q11).<br>• could also send a message on group chat (C113, Q6a).<br>• When your on Teams call it's difficult to concentrate (C114, Q3a).	
		**3.1.2 Enhancing platform experience**	**3.1.2.1 Issues with Teams link**<br>• had issues logging on initially when enrolled onto two courses, now enrolled onto a third course and IT issues have resolved (C101, Q10).<br>• A few technical issues no and again. Still managed to do - slides didn't work sometimes (C109, Q10).<br>• Teams is probably best way really (C109, Q11).<br>• the only issue I had it automatically let me in and not into the waiting room as I work for the NHS. sometimes if lots of people had the cameras on and microphones it slowed the connection down (C113, Q10).<br>• Yes I did. click on link - it would give 3 ways to open. even when my son was with me he couldn't get in. once it made me poorly because I couldn't get in (C214, Q10).<br>• i couldn't always access the link (C222, Q10).<br>• Difficulty with link so couldn't join (C113, Q4).<br>• First on smart phone first 3. Then on tablet on 4th - that's was better. **I could see more people - which was important* and I could use the functions. Better experience. After that couldn't get on link, tried and tried and then gave it up as a bad job (C114, Q10).<br>• had to ring up several times for the link as hadn't received it. struggled to use it in general (C103, Q10).<br>• yes, had to ring up several times for the link as hadn't received it. struggled to use it in general (C103, Q10).<br>• I rang following day to long covid people. Tried resending link didn't work (C114, Q10).
			**3.1.2.2 Solutions to link issue**

			• Zoom might have been easier for me rather than using all our Teams Never straight forward (C2 14, Q10).<br>• would have been better if there was lesson on how to use Microsoft teams first (C103, Q6a).<br>• i couldn't always access the link. i would appreciate if the link was emailed out each week or a reminder. (C222, Q10).<br>• Having content on youtube so those who could not attend the group could access video content (S12, Q6).<br>• to give them the option of receiving the slides each week in a paper copy or email if they prefer (S16, Q6).
	**3.2 Confidence**	**3.2.1 Hardware**	**3.2.1.1 Computer literacy**<br>• I'm not very good with computers. (C218, Q11).<br>• At first but got the hang of it (C221, Q10).<br>• Not able to comment as did not attend any due to lack of IT skills. Asked if let HCP know of difficulty and said had not (C229, Q13a).<br>• Not able to comment as did not attended any due to lack of IT skills (C229, Q10, Q8b, Q8a, Q7a, Q6a, Q5a).<br>• No IT knowledge (C229, Q4).<br>• Occasionally with ipad (C232 Q10).<br>• had used skype and zoom but not teams (C101, Q10).<br>• Not able to comment as did not attended any due to lack of IT skills. Husband was away and not confident with internet. Online my husband wasn't able to get it on his email (C229, Q3a, Q2a).<br>• didn't know how to log on to get onto Teams or what buttons (C218, Q10).
			**3.2.1.2 IT/Technical issues**<br>• struggled to participate due to use of MS teams - struggling to work out how to contribute to discussions (C103, Q6a).
			**3.2.1.3 Wi-Fi**<br>• Only once due to poor internet (C211, Q10).<br>• One session - problem with my internet at home (C213, Q9a).

Main Themes	Main Sub-Themes	Sub Theme	
			• felt the course would've been relevant for me if i'd been able to access it (C218, Q14).
	**3.3 Delivery**	**3.3.1 Mass Delivery**<br>• It gives them more intensive input than we can otherwise provide (S4, Q3).<br>• To deliver generic information to a mass audience (S8, Q3).<br>• Provide therapy interventions to a larger group of people (S14, Q3).<br>• can reach a wide audience at once (S6, Q4).<br>• too many are just put on the course but have no genuine chance of completing (S11, Q6).	
		**3.3.2 Multi-Disciplinary Approach**<br>• an MDT approach (S1, Q4).<br>• multi discipline (S3, Q4).<br>• variety of clinicians presenting (S13, Q6).<br>• To deliver intervention from multiple professions in a safe and condensed way (S2, Q3).	
	**3.4 Slides**	**3.4.1 Availability of Slides**<br>• Slides only unable to attend due to work and times of sessions (C215, Q6a).<br>• I was able to look back at slides and reuse them to help myself (C105, Q5a).<br>• Good to get the slides after and read through (C116, Q5a).<br>• did not attend the course, reviewed slides after (C218, Q2a).<br>• Found slides for causes of breathlessness useful even though didn't make session (C117, Q3a).<br>• accessed slides after the course as did not attend the actual course. all the information was really relatable (C218, Q5a).<br>• like the fact the slides were emailed after so I could refer back to the session (C236, Q5a).<br>• slides following the session to enable them to reflect back/ go back to review the information (S1, Q4).<br>• receiving the slides either via email or a paper copy so they have the same information (S16, Q5).	
		**3.4.2 Availability of Voice Recordings**<br>• don't yet have the slides with the voiceover to send to patients (S1, Q5).<br>• have the content accessible on a video platform as we can only send the slides with no sound (S5, Q5).<br>• Rather than just a voiceover to add a person talking through the slides. Recordings available for people to access in free time eg on a youtube channel (S3, Q6).<br>• Patients to have access to the programme and the voiceover to allow them to reflect back on (S1, Q6).<br>• Patients being able to access the content (videos, with sound etc (S4, Q5).	
		**3.4.3 Appearance**	**3.4.3.1 Professionalism**<br>• presentations differ in appearance and could look more professional (S1, Q5).<br>• some of the terminology and avoid terms such as 'correct' and 'incorrect' etc (S17, Q5).<br>• less busy and wordy slides (S2, Q6).<br>• More professional appearance: having input from graphic designer to design the slides and how they look (S3, Q6).

**4 Group Dynamics**	**4.1 Interaction**	**4.1.1 Positives**	**4.1.1.1 Story telling – sharing experience**<br>• I thought that was good. Other people with similar problems (C114, Q6a).<br>• felt that it made it real and nice to hear other people's experiences (C113, Q6a).<br>• Overall yes. Big thing with long covid is the isolation - you realise someone else is verbalising what you are feeling real value in that ! (C2 28, Q6a).<br>• to have that discussion with a support network who had understanding and in the same position was really healing and reassuring (C234, Q6a).<br>• It was nice to hear that people were struggling and had symptoms (C116, Q2a).<br>• Group dynamic, patients not feeling alone (S6, Q4).<br>• Members of the group feel comfortable to come forward and share their experiences (S13, Q4).<br>• Positive patient stories/journey examples (S15, Q5).<br>• To allow patients to share their post COVID journey's with others- peer discussion (S1, Q3).<br>• Support from other patients so they can see they are not alone (S3, Q3).<br>• how Long Covid is a shared experience by many other people - just like them (S5, Q3).<br>• To learn from peers, things that have helped them to manage symptoms and recovery (S5, Q3).<br>• Peer support (S6, Q3).<br>• To provide some form of peer support through a wider network (S10, Q3).<br>• To provide a safe place for those recovering from long Covid to share and obtain peer support (S12, Q3).
			**4.1.1.2 Missing shared experience**<br>• Everyone can talk, different people who have gone through the same thing, at different stages. People may contact one another and maybe meet up (C114, Q11).<br>• I miss doing the course having discussions with other people with long covid and having

			discussions about how others have managed their long covid. it was really helpful (C113, Q15).
			**4.1.1.3 Less helpful**<br>• Perhaps more of a discussion would be helpful. So you getting others peoples take on it (C109, Q3a).<br>• felt that due to pre-existing condition that this was not needed. (C101, Q6a).<br>• Could be very repetitive weak after week - same people (C109, Q6a).<br>• I found very little of the end of session discussions to be relatable to my situation. (C2 17, Q6a)<br>• I feel the peer support is important and I didn't really get that from the session (C216, Q7a).<br>• Some patients do not engage with clinicians and don't want to show their camera especially if carrying out interactive part of breathing retraining so unsure if patients are even listening and following interactive parts of course (S7, Q5).<br>• Being on teams is another barrier to reducing that interaction with some patients as may be easier to interact with patients in face to face (S7, Q5).
			**4.1.1.4 Group size/dynamics impacts on interaction**<br>• Generally, I found that useful however group size an issue (C216, Q6a)<br>• Too big (C216, Q7a).
		**4.1.2 Interaction: enablers, solutions & opportunities**<br>• If groups too big use break out rooms. and rotate between (C216, Q7a)<br>• If groups need to big use break out rooms. and rotate between (C216, Q7a)<br>• Virtual works well - introduction would be good for members to extra 15 mins at the end - 'how is everybody?' does anyone have any feedback to share... a bit of community spirit Face to face would be difficult...who is in the group. Mixed group would be challenging (C232, Q11).<br>• Perhaps partners being invited on it would be helpful. They are still learning...like with brain fog if your partners there they ask questions (C109, Q3a).<br>• To provide a platform that is easily accessible for patients with long covid (S10, Q3).<br>• a wider network on a virtual platform (S10, Q3).	

		• Members can also use chat function if they do not wish to speak (S13, Q4).	
	**4.2 Participation**	**4.2.1 Benefits**	**4.2.1.1 No longer alone**<br>• Because long covid thinking no one understood or going through it. sense of being in it together with others. some useful tips...useful to hear (C2 27, Q6a).
			**4.2.1.2 Sharing the struggle**<br>• I did recognise that there were people worse than me. I was able to share my experience (C105, Q3a).<br>• It was nice to hear that people were struggling and had symptoms (C116, Q2a).<br>• it was good to hear other people's experiences, some of the suggestions made by other people were really good. (C2 25, Q6a)
			**4.2.1.3 Flexibility of participation**<br>• it was good there was no pressure to talk (C113, Q6a).<br>• Good to have a chance for people to bring up anything.
			**4.2.1.4 Opportunity for Questions**<br>• ms teams - liked being able to ask questions (C111, Q11).<br>• questions live with a clinician (S2, Q4).<br>• provides a platform to ask questions specific to their symptoms in relation to course content (S7, Q4).<br>• Patient seem to like the Q&A/peer support at the end of each session (S8, Q4).<br>• questions and answers part I think is great and get good feedback from patients about this (S16, Q4).<br>• Sometimes difficult to answer all questions proposed due to time limits (S7, Q5).
			**4.2.1.5 Value of Peer Support**<br>• I think I contribute to Mental wellbeing (C117, Q3a).<br>• Overall good having time to talk to other and life course (C117, Q6a).<br>• I think its a really good idea to have - outlet to talk about. Knowing support is there. (C2 26, Q15)<br>• listening to discussion was good (C117, Q3a).

			• opportunity for peer support (S2, Q4).<br>• Peer discussion element often reported as valuable by patients (S9, Q4).<br>• the groups are too big for any meaningful peer discussion (S3, Q5).<br>• Limited opportunity for patients to have that peer support that they need - enabling patients to speak with each other but this is something to be developed as an add on to the course (S7, Q5).
		**4.2.2 Barriers**	**4.2.2.1 Individual level**<br>• people and myself were reluctant to give information out due to embarrassment (C113, Q3a).<br>• that one of their most limiting factors is fatigue (S10, Q3).<br>• This can be quite difficult for those who have fatigue (S1, Q5).
			**4.2.2.2 Group level**<br>• I had no idea how many people were on it. Hardly anyone had camera on. I used it to do my breathing exs and to lay down - so kept my camera off. (C214, Q7a)<br>• everyone had their camera off so it didn't bother me or didn't matter how many people were there (C225, Q7a)<br>• in general the size of the group worked.(C236, Q7a).<br>• I don't think the group size really affected what was going on because it was online. it might be beneficial for a bigger group so more people discuss (C234, Q7a).<br>• Couldn't really tell - camera off (C232, Q7a).<br>• not every session was taken by the same staff, so because it was presented by different staff each week they weren't aware the same participants were dominating the discussion. it was difficult to join in the conversation sometimes (C236, Q6a).
		**4.2.3. Enhancing participation**	**4.2.3.1 Individual**
			**4.2.3.2 Group**<br>• I think I felt that I didn't contribute because I found it impersonal. So you don't share with people. Would have been nice if people could give a bit of

			their hx, how they are now and how they have progressed. Sometimes there was maybe 3 or 4 people who contributed others less so. You don't know people (C2 13, Q6a)<br>• Too big just because if people wanted to talk at the end might run out of time. Although bigger group people might chip in more ? (C2 13, Q7a)<br>• LC is quite isolating, the only other people I know are on the Tv. Building those relationship is probably helpful. (C2 15, Q11)<br>• I would have liked it if people had participated a bit a more or it was a bit a longer. (C2 22, Q6a).
	**4.2 Facilitation: Presenter/Delivery**<br>• I think they all made us feel welcome in their friendliness even with use of computer. That showed you that we are all the same (C105, Q15).<br>• also good therapists were willing to expand on bits of the presentation if needed (C113, Q6a).<br>• Just that the people who delivered the course were really good, so patient and professional and good at moving things on. Sharon was really good. No question was too left field. If they couldn't answer a question they would get back to that person (C228, Q15).<br>• everyone was really helpful and friendly (C236, Q15).<br>• the clinicians were really helpful to provide insight. I felt this was really powerful. (C234, Q6a).<br>• I know it was hard as I've online Mental Health things - smaller groups. Facilitators to keep everyone involved (C116, Q2a).<br>• I thought it was. The only problem was that one lady who took over the show (C116, Q6a).		

	• Some lacked a bit of content with talking - then sometimes it would be better (C109, Q3a).<br>• It varies from session to session. Sometimes we ran out of time (C117, Q6a).<br>• not every session was taken by the same staff, so because it was presented by different staff each week they weren't aware the same participants were dominating the discussion. it was difficult to join in the conversation sometimes (C2 36, Q6a).		
**5 Individual Factors**	**5.1 Pre-Existing Conditions**<br>• has MS felt the subsequent sessions were aimed at people who were well and then had long covid, whereas I feel I have already been through that process (C101, Q2a)<br>• felt that due to pre existing condition that this was not needed (C101, Q6a).<br>• cerebellum atrophy and ataxia struggles with progressive exercise and activity and soon get tired. trapped nerve stopped meant struggling to move as well and mental wellbeing (C103, Q8b).<br>• Life I have to work, doing my dissertation, everyday things I have to do LC advice goes against my brittle asthma advice from my other Drs which can be a barrier C2 15, Q8b)		
	**5.2 Long COVID Symptoms**	**5.2.1 Impacts of long COVID symptoms**	**5.2.1.1 Fear of return Long COVID symptoms/uncertainty**<br>• it has improved since (C111, Q13).<br>• progressing exercise and activity - felt this was challenging due to fatigue and breathlessness (C113, Q3a).

			• Having the energy to fit in breath ex’s and return to ex’s (C2 116, 8b)<br>• Some useful tools but worry that this will re-occur (C2 11, Q13a)<br>• reduce risk of exacerbation (S9, Q3).
			**5.2.1.2 Stage of Long COVID journey**<br>• Although selecting 'No' for the question above, it is not a reflection on the quality of the information presented. It is a reflection of my own particular circumstances; the overall course met with my expectations. (C2 17, Q13a)<br>• giving people information to understand their experience better (S4, Q3).
	**5.3 Individual care needs**	**5.3.1 Adaptability**<br>• not flexible or adaptive to individuals eg some people will want more info quicker and others a slower pace (S3, Q5).<br>• It doesn't fit well as a one size fits all (S4, Q5).<br>• people would benefit from this flexibility so they can catch up, or go at a different pace (S4, Q5).<br>• Not always dynamic enough (S5, Q5).<br>• Having to cover all information to accommodate a wide audience so some information may not be relevant to some patients (S7, Q5).	
**Main Themes**	**Main Sub-Themes**	**Sub Themes**	
**6 Internal Change**	**6.1 Self-Reflection**	**6.1.1 Positive reflection**<br>• I’d only just got myself a computer my brother was amazed I downloaded Teams. I wanted to understand it myself from being up and down (C105, Q10).<br>• Yes I think it has given me things to help myself (C105, Q13).<br>• I had done quite a lot of time to research long covid, watch webinars etc and put together some plan of attack (C117, Q5a).<br>• absolutely, to understand what's going on in my body and knowing how I can manage this is the key; diet was really useful (C234, Q13a).<br>• It made me realise I was doing well compared to others on the call. I'm doing quite well actually. When I was feeling unwell again (C105, Q6a).<br>• the discussions really impacted on me (C113, Q6a).<br>• You can get to stage that you think its all in your head (C116, Q3a).<br>• You got used to contributing (C116, Q6a).	

		• I thought what that meant - gave me a measure of it. It made me think more about my energy (C116, Q8a).<br>• None just me putting my mind to doing it ! (C2 13, Q8b)	
		**6.1. 2 Negative reflection**<br>• Exs & Act: made me feel bad about myself - muscle loss made me panic about that...doesn't mean people don't to know (C117, Q3a).<br>• it certainly helped, I feel I have a lot of problems due to covid that could not be helped by the virtual course (C103, Q8a).<br>• I found more things out after finishing the course (C109, Q5a).	
	**6.2 Self Efficacy**	**6.2.1 Recognising self-efficacy**	**6.2.1.1 Supporting self-efficacy**<br>• sometimes you need that validation (C215, Q2a).<br>• symptoms diary helped me understand what I can do and helped me build my activity (C225, Q2a).<br>• I suppose it has - its done things and my helped with my own expectations. Once those are in place - I'm mentally in tune. There are always things you can try and give it go. you need to be open to the idea of getting better (C228, Q13a).<br>• absolutely, to understand what's going on in my body and knowing how I can manage this is the key; diet was really useful (C234, Q13a).
			**6.2.1.2 External control**
	**6.1 Self-Management**	**6.3.1 Self-management learnings**	**6.3.1.1 Pacing**<br>• working within the boom and bust cycle ensured I could do the skills. (C224, Q8b).<br>• I now meditate 3 times a day at work. Reduced workload at work. I have learnt to be a bit more selfish so that I am not too tired. I do breathing exercises. I monitor myself at work and I now have some energy outside of work. I feel that managing my energy better is down to the virtual course (C113, Q6a).<br>• I did try pacing definitely, website for relaxing (C213, Q8a).<br>• Pacing myself. Plan more each day. Take each day as it comes (C221, Q13).

			• I learned to say no after crashing and burning (C109, Q8a).<br>• the boom and bust cycle and breathing exercises and physical activity were really helpful for me. breathing techniques were key for my POTS. (C234, Q8a)
			**6.3.1.2 Breathing**<br>• breathing exercises were helpful (C103, Q8a).<br>• able to reuse when required (C105, Q8a).<br>• able to participate breathing exercises (C111, Q8a).<br>• Its provided some skills to manage it (C116, Q13).<br>• breathing exercises, ive been timing how long I can exercise for and its about 6 minutes. (C222, Q8a).<br>• breathing techniques were key for my POTS. (C234, Q8a)<br>• the slides helped me learn the breathing techniques. it helped me learn a completely new way of breathing (C218, Q13a).<br>• Really good resources/detailed information Breathlessness one was really good, empowering, positive (C232, Q13a).<br>• breathing exercises (C101, Q8a).<br>• had significant breathlessness and found the breathing sessions helped. generally the sessions were all very good (C103 Q2a).<br>• Causes of Breathlessness. Re-gone over this and that has helped to use as self-correct (looked in mirror) (C105, Q3a).<br>• able to participate breathing exercises (C111, Q8a).<br>• causes of breathlessness really helped with some techniques to help manage these (C113, Q2a).<br>• felt that the breathing retraining was already covered in the first one. mental wellbeing felt repetitive of things I already knew (C111, Q3a).<br>• I think reiterating the importance of breathing exs was good (C216, Q8a).<br>• breathing exercises - I was having panic attacks so they really helped steady my breathing (C225, Q2a).

			• i've definitely been doing the breathing exercises, I was keeping a symptom diary prior to the course.(C225, Q13a).<br>• they were the biggest areas I was having issues (C236, Q2a).
			**6.3.1.3 Diet and nutrition**<br>• I got to point that due to fatigue I wasn’t eating properly – eating the wrong food. I batch cook to avoid this (C105, Q3a).<br>• Some of them - hydration/nutrition/screen breaks Sleeping health good Pacing & exs have improved: HR, BP all improved in time (C232, Q8a).<br>• absolutely, to understand what's going on in my body and knowing how I can manage this is the key; diet was really useful (C234, Q13a).<br>• Some of the information in the 'Diet' session spurred further changes to my diet that have been beneficial. (C217, Q2a).<br>• Nutrition - difficult to get good nutrition when I'm poorly I've made sure I've had a good eating day (C214, Q8b).<br>• Diet. I revisited this and it had helped with preparing food etc and getting healthy meals in (C105, Q3a).<br>• diet was really useful (C234, Q13a).<br>• diet session with supplements was interesting (C232, Q6a).
			**6.3.1.4 Measuring improvement**<br>• 75% yes I can mange more (C109, Q14).<br>• it has improved since (C111, Q13).<br>• I thought what that meant - gave me a measure of it. It made me think more about my energy (C116, Q8a).<br>• when I started the course I physically couldn't get around, by the end of the course I had returned to work. (C236, Q8a).
			**6.3.1.5 Goal setting**<br>• Maybe not 100% but contributed Having some personal goals in d/w hcp and course would be helpful (C216, Q13).
			**6.3.1.6 Fatigue**

			• fatigue management and causes of breathlessness really helped with some techniques to help manage these (C113, Q2a).<br>• felt fatigue management and sleep and relaxation were least useful as already competent at managing due to existing condition (C101, Q3a).<br>• Fatigue management - I liked the way it was split into 2 - maybe be more specific at the beginning that will be returning to this (C2 28, Q2a).
		**6.3.2 Mechanisms for knowledge gains**<br>• I found more things out after finishing the course (C109, Q5a).<br>• peoples understanding of what long covid is (C113, Q8b).<br>• Diet - I knew most of that ironically being overweight ! Sleep & relax/mental wellbeing - prevs experience Useful in their own way everything had value (C215, Q3a).<br>• Sometimes I plan for it a special occasion and then know I'll need to rest for a few days. I avoid that on a day to day basis and have reduced my working hrs to help with fatigue (C215, Q8a).<br>• The course content and depth of information was broadly what I expected. It was however a useful aggregation of information, but with only a limited amount pertinent to my situation (C217, Q3a).<br>• everything you've covered is really useful - it provides everything you need to manage long covid (C227, Q13).<br>• to facilitate patient's self management (S2, Q3).<br>• help self management of long covid symptoms (S16, Q3).<br>• how to apply what is being learnt (S6, Q6).	
		**6.3.3 Advancing self-management skills**	**6.3.3.1 Combining techniques**<br>• support the rest of the therapy input delivered in the service(S16, Q3).<br>• allow 1:1 clinic time to be used for more individualised therapy (S8, Q3).<br>• to learn more to aid their rehabilitation (S11, Q4).<br>• and they are expected to try things out to then discuss at the reviews; it is not a passive course (S4, Q6).

			**6.3.3.2 Alternative Therapies**<br>• I'd started doing Yoga Nidra (C105, Q3a).<br>• Well being/relaxation – Yoga Nedra and mindfulness Breathing (C105, Q8a).<br>• I started Yoga since doing the course – which helps (C109, Q8b).<br>• Shared Harmony would have been good (C109, Q14).<br>• *to share things that are helping like alternative treatments. (C234, Q14).*
			• **6.3.3.3 Strategies**<br>• To provide tools and strategies to support patients in self managing their ongoing symptoms (S1, Q3).<br>• Advise on self management strategies (S3, Q3).<br>• To start to employ self management strategies for recovery (S5, Q3).<br>• help patients engage with self management techniques (S6, Q3).<br>• provide a patient with the tools to enable them to self manage their symptoms (S7, Q3).<br>• help them to develop strategies to address managing these symptoms (S7, Q3).<br>• support with the self-management (S10, Q3).<br>• To provide useful interventions (S13, Q3).<br>• provide self management skills (S14, Q3).<br>• a range of strategies and techniques to self manage symptoms (S15, Q3).<br>• self management strategies (S17, Q3).<br>• Breathing information Session on diet and nutrition has good examples of how to make changes (S15, Q4).<br>• Exercise/activity progression to be covered in more detail (s12, Q6).<br>• have as many practical tips and advice as possible (S17, Q6).<br>• shows you different techniques to help yourself (C109, Q2a).<br>• One thing that stuck out in my mind. Using your energy an using it like spoons (C116, Q8a).<br>• Definitely able to put into practice even if its sometimes counterintuitive - occasionally I'll get it wrong and end up in bed. Sometimes I plan for it a

			special occasion and then know I'll need to rest for a few days. I avoid that on a day to day basis and have reduced my working hrs to help with fatigue (C215, Q8a).<br>• breathing exercises - I was having panic attacks so they really helped steady my breathing. fatigue - symptoms diary helped me understand what I can do and helped me build my activity (C225, Q2a).<br>• Practical session week 9. something I could actually do !it was quite powerful actually, practical, a doing thing I liked that ! (C228, Q2a).<br>• there were pretty good. the diet one I missed first half but thought useful regarding supplements etc (C227, Q2a)<br>• when I started the course I physically couldn't get around, by the end of the course I had returned to work (C236, Q8a).
		**6.3.4 Knowledge of Symptoms**<br>• plus an overview of Long COVID and understanding of what it does to the body (S10, Q3).<br>• education on the theories of why symptoms present (S7, Q3).<br>• To improve a patient's understanding of Long COVID and potential causes for their ongoing symptoms (S8, Q3).	
	**6.4 Acceptance of condition**	• Fatigue is one of my biggest symptoms. some times you need that validation (C2 15, Q2a)<br>• I was a fit person, but now I have fatigue and muscle pain. (C2 22, Q8b)<br>• it has certainly given me something, everyone is learning from Long covid and i feel no one has a magic wand. it hasn't cured me but it has given me some direction. (C2 22, Q8a)	
